# Increase of Short-Term Heart Rate Variability Induced by Blood Pressure Measurements during Ambulatory Blood Pressure Monitoring

**DOI:** 10.1155/2017/5235319

**Published:** 2017-04-03

**Authors:** Attila Frigy, Annamária Magdás, Victor-Dan Moga, Ioana Georgiana Coteț, Miklós Kozlovszky, László Szilágyi

**Affiliations:** ^1^Department of Internal Medicine IV, University of Medicine and Pharmacy of Tîrgu Mureș, Tîrgu Mureș, Romania; ^2^Department of Cardiology I, Victor Babeș University of Medicine and Pharmacy, Timișoara, Timișioara, Romania; ^3^BioTech Research Center, Óbuda University, Budapest, Hungary; ^4^Faculty of Technical and Human Sciences, Sapientia Hungarian University of Transylvania, Tîrgu Mureș, Romania; ^5^Department of Control Engineering and Information Technology, Budapest University of Technology and Economics, Budapest, Hungary

## Abstract

*Objective.* The possible effect of blood pressure measurements per se on heart rate variability (HRV) was studied in the setting of concomitant ambulatory blood pressure monitoring (ABPM) and Holter ECG monitoring (HM).* Methods.* In 25 hypertensive patients (14 women and 11 men, mean age: 58.1 years), 24-hour combined ABPM and HM were performed. For every blood pressure measurement, 2-minute ECG segments (before, during, and after measurement) were analyzed to obtain time domain parameters of HRV: SDNN and rMSSD. Mean of normal RR intervals (MNN), SDNN/MNN, and rMSSD/MNN were calculated, too. Parameter variations related to blood pressure measurements were analyzed using one-way ANOVA with multiple comparisons.* Results.* 2281 measurements (1518 during the day and 763 during the night) were included in the analysis. Both SDNN and SDNN/MNN had a constant (the same for 24-hour, daytime, and nighttime values) and significant change related to blood pressure measurements: an increase during measurements and a decrease after them (*p* < 0.01 for any variation).* Conclusion.* In the setting of combined ABPM and HM, the blood pressure measurement itself produces an increase in short-term heart rate variability. Clarifying the physiological basis and the possible clinical value of this phenomenon needs further studies.

## 1. Introduction

Ambulatory blood pressure monitoring (ABPM) coupled with Holter ECG monitoring (HM) is more and more used in clinical practice, mainly in hypertensive patients, for diagnosing arrhythmias and ischemic events and for establishing their relationship with blood pressure variations [1]. On the other hand, beyond its classical role, HM could provide useful information on the cardiovascular autonomic control by heart rate variability (HRV) analysis. HRV represents the variations of the normal sinus rhythm, which are related to the fluctuations of the cardiac autonomic tone [2]. Short-term (up to 5 minutes) HRV consists mainly of high frequency (HF, 0.15–0.4 Hz), vagally mediated oscillations, containing also a low frequency (LF, 0.04–0.15 Hz) component related to baroreflex activity and sympathetic nervous modulation. LF/HF power ratio is conventionally used for the characterization of the cardiac sympathovagal balance. Long-term (e.g., 24 hours) variability incorporates high, low, very low (VLF, 0.0033–0.04 Hz), and ultralow (ULF, <0.0033 Hz) frequency components. HRV can be measured by statistical (time domain), spectral (frequency domain), and nonlinear methods, with the first being the most robust to ectopic beats and registration artifacts. Time domain parameters of short-term HRV are strongly correlated among them and with spectral measures (HF and LF components) [2–5].

HRV has a clinically important significance: decreased HRV is an established negative prognostic marker in many cardiac and noncardiac conditions [6–10]. Also, cardiac autonomic neuropathies (e.g., diabetic) could be diagnosed on the basis of blunted HRV [11].

In the context presented above, we proposed to examine the interaction of HRV with blood pressure measurement itself, a phenomenon which was not studied before in the Holter literature. We considered that finding a significant interaction could be of interest both methodologically and clinically.

## 2. Methods

As a part of routine exploration, 25 ambulatory hypertensive patients (14 women and 11 men, mean age: 58.1 years) underwent 24-hour combined ABPM and HM for diagnosing possible arrhythmic events related to blood pressure variations. All subjects gave written informed consent, and the study was approved by the local Ethical Committee, according to the International Ethical Guidelines and Declaration of Helsinki.

Blood pressure measurements were set at 15 minutes during the whole 24-hour monitoring. For every blood pressure measurement, 2-minute ECG segments (before, during, and after blood pressure measurement) were analyzed to obtain time domain parameters of HRV: SDNN (standard deviation of normal RR intervals) and rMSSD (root mean square of successive differences between normal RR intervals). MNN (mean of normal RR intervals), SDNN/MNN, and rMSSD/MNN (heart rate corrected, normalized parameters) were extracted, too. HRV parameters were calculated using standard formulas [2, 3]. SDNN reflects global variability, while rMSSD is a measure of vagally mediated heart rate oscillations. In case of short-term recordings, SDNN and rMSSD are strongly correlated [2, 4].

The measurements were performed using the card(X)plore combined 24-hour blood pressure monitor (ABPM with 3-channel HM, Meditech Ltd., Hungary) with 600 Hz, 12-bit sampling characteristics. First, the moments of blood pressure measurements were identified (start points) on the Holter recording. Then, after manual editing and corrections, the RR series data of the 2-minute segments were exported to Matlab R2015a software for HRV calculations and statistical analysis. The 2-minute interval was chosen because of the duration of blood pressure measurement (cuff inflation and deflation).

All 2-minute ECG segments with ectopic beats and/or artifacts affecting 10% of the recording were excluded from analysis.

Blood pressure measurements occurring during nonstationary ECG signal segments were detected according to the following protocol:

(1) For each blood pressure measurement, we computed the SDNN parameter value within the 2-minute intervals occurring before (SDNN_before_), during (SDNN_during_), and after (SDNN_after_) the measurement.
(2) We defined the temporary stationarity index (TSI) as
  (1) $$\begin{array}{c} \text{TSI}=\frac{\max\left({\text{SDNN}}_{\text{before}},{\text{SDNN}}_{\text{during}},{\text{SDNN}}_{\text{after}}\right)}{\min\left({\text{SDNN}}_{\text{before}},{\text{SDNN}}_{\text{during}},{\text{SDNN}}_{\text{after}}\right)}. \end{array}$$
(3) Blood pressure measurements having TSI > 1.5 were excluded from the analysis due to the temporary nonstationary behavior of the ECG signal.

The variations of HRV parameters related to blood pressure measurements (obtained for 24 hours, daytime, and nighttime) were analyzed using one-way ANOVA with multiple comparisons (significant change if *p* < 0.05).

## 3. Results

From the total of 2461 measurements, after signal stationarity validation, 2281 measurements (1518 occurring during the day and 763 during the night) were included in the analysis.  Table 1 presents the mean values (±standard deviation) of MNN and HRV parameters (also those normalized with heart rate: SDNN/MNN and rMSSD/MNN) before, during, and after blood pressure measurements. SDNN and SDNN/MNN showed a constant (for the 24-hour, daytime, and nighttime values) and significant change related to blood pressure measurements: increasing during measurements and decreasing after them. This behavior was not influenced by the underlying heart rate. For rMSSD, the tendency was the same but was not statistically significant.  Table 2 presents *p* values obtained from the comparison of HRV parameters before, during, and after blood pressure measurements.

**Figure 1:**
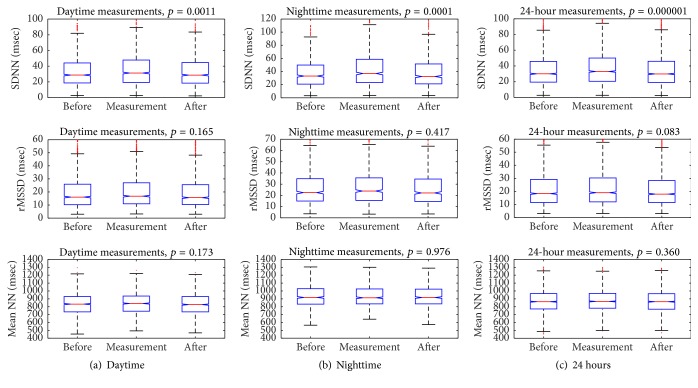
Box-and-whiskers plots illustrating the behavior of HRV parameters related to blood pressure measurements. The red line represents the median value, the blue box shows the 25%–75% interquartile range (IQR), whiskers show +/− 1.5 × IQR distance from the edges of the boxes (in case of symmetrical distribution, this means median +/− 2 × IQR), and red points show the outliers.

The behaviors of parameters related to blood pressure measurements (obtained for 24 hours, daytime, and nighttime) and the corresponding *p* values resulting by applying ANOVA are presented graphically in Figure 1 (box-and-whiskers plots). The graphics illustrate that only SDNN had a significant change (for the 24-hour, daytime, and nighttime values).

## 4. Discussion

Combined devices of ABPM with HM are popular nowadays, mainly for assessing blood pressure related changes of heart rate (premature beats, arrhythmias), not only in hypertensive patients. HRV, a measure of cardiac autonomic balance, is analyzed routinely by these devices [12]. Accurate determination of various HRV parameters is essential for reliable risk evaluation in diverse clinical conditions including ischemic heart disease, cardiomyopathies, and heart failure and hypertension as well. There are numerous data in the literature regarding the usefulness of HRV analysis in the risk stratification in these settings [2, 3, 6].

Studying the possible impact of blood pressure measurements per se on short-term HRV, we found a significant increase of total variability, reflected by SDNN, during the blood pressure measurements. This effect was not related to the change in heart rate and was not associated with the increase of the more vagally mediated, beat-to-beat based rMSSD. However, an important vagal influence in the determination of SDNN in the case of short-term recordings is well known [2–4]. The possible explanations of the observed phenomenon could be the following: (1) during the day, the patients assume a resting position when measurements start, which can enhance vagal influences, and (2) during the blood pressure measurement, by compressing the brachial artery, the peripheral vascular resistance increases, which could enhance vagal modulation via baroreflex mechanisms. These supposed mechanisms or other different explanations have to be demonstrated and clarified by further studies.

Our finding could have a clinical relevance from many points of view: (1) existence of a significant influence on the values of SDNN calculated from short-term segments of Holter recordings, especially in the case of noncontinuous blood pressure measurement triggered ECG recordings and (2) the possible application of the phenomenon in improving risk stratification. To clarify the latter aspect, a study is needed on the characteristics of patients who show/do not show this behavior of HRV. The lack of the increase in HRV during blood pressure measurement could serve as a new criterion for risk stratification, reflecting an impaired baroreflex control. It could also be of clinical interest to examine the relationship of the observed phenomenon with the measured individual blood pressure values and with diverse characteristics of the 24-hour blood pressure profile.

## 5. Conclusion

During combined (ABPM + HM) monitoring, blood pressure measurements per se induce a perturbation of HRV, provoking increase of global variability during measurements. This phenomenon could have methodological and clinical implications when evaluating patients undergoing this kind of monitoring. However, clarification of the physiological background and the possible clinical value of this phenomenon needs further studies.

## Figures and Tables

**Table 1:** Mean values ± standard deviation of Mean NN (MNN) and HRV parameters before (B), during (M), and after (A) blood pressure measurements. Normalized values represent the correction with heart rate (SDNN/MNN and rMSSD/MNN). The *p* values are the results of one-way ANOVA tests; significant differences (*p* < 0.05) are written in italic; *p* values marked with *∗* refer to normalized parameters.

	2-Minute interval	Mean NN (msec)	*p* value	SDNN (msec) Normalized (%)	*p* value	rMSSD (msec) Normalized^*∗*^ (%)	*p* value
24 hours	B	869 ± 143	0.360	35.68 ± 23.76	*<10* ^−*6*^ *<*10^−6^^*∗*^	22.52 ± 15.24	0.083 0.058^*∗*^
				4.11 ± 2.65		2.59 ± 1.75	
	M	874 ± 140		39.47 ± 27.04		23.53 ± 16.40	
				4.52 ± 3.10		2.69 ± 1.88	
	A	868 ± 143		35.85 ± 23.98		22.35 ± 15.51	
				4.13 ± 2.76		2.58 ± 1.79	

Daytime (06:00–22:00)	B	834 ± 136	0.173	33.98 ± 21.60	*0.0011* *0.0044*^*∗*^	19.93 ± 13.25	0.165 0.207^*∗*^
				4.08 ± 2.59		2.39 ± 1.59	
	M	841 ± 133		36.46 ± 23.51		20.83 ± 13.95	
				4.34 ± 2.80		2.48 ± 1.66	
	A	832 ± 136		33.71 ± 21.26		19.83 ± 13.49	
				4.05 ± 2.55		2.38 ± 1.62	

Nighttime (22:00–06:00)	B	940 ± 130	0.976	39.14 ± 26.03	*10* ^−*4*^ *<*10^−4^^*∗*^	28.12 ± 18.64	0.417 0.260^*∗*^
				4.16 ± 2.77		2.99 ± 1.98	
	M	939 ± 130		45.80 ± 33.74		29.33 ± 20.54	
				4.88 ± 3.59		3.12 ± 2.19	
	A	938 ± 131		40.29 ± 29.41		27.79 ± 19.01	
				4.29 ± 3.13		2.96 ± 2.03	

B, 2-minute interval ending when the blood pressure measurement begins; M, 2-minute interval starting when the blood pressure measurement begins; A, 2-minute interval starting two minutes after the moment when the blood pressure measurement begins.

**Table 2:** *p* values obtained by one-way ANOVA tests comparing HRV parameters (before (B) versus during (M), during (M) versus after (A), and before (B) versus after (A) BP measurements). Significant differences (*p* < 0.05) are written in italic; *p* values marked with *∗* correspond to normalized parameters.

	SDNN			rMSSD		
	B versus M	M versus A	B versus A	B versus M	M versus A	B versus A
24 hours	*<10* ^−*5*^	*<10* ^−*5*^	0.883	0.091	*0.037*	0.682
	*<*10^−5^^*∗*^	*<*10^−5^^*∗*^	0.752^*∗*^	0.059^*∗*^	*0.030* ^*∗*^	0.760^*∗*^
Daytime	*0.0008*	*0.0036*	0.678	0.141	0.077	0.774
	*0.0078* ^*∗*^	*0.0032* ^*∗*^	0.776^*∗*^	0.145^*∗*^	0.112^*∗*^	0.884^*∗*^
Nighttime	*<10* ^−*4*^	*0.0015*	0.506	0.336	0.214	0.759
	*<*10^−4^^*∗*^	*0.0007* ^*∗*^	0.395^*∗*^	0.215^*∗*^	0.130^*∗*^	0.763^*∗*^

B, 2-minute interval ending when the blood pressure measurement begins; M, 2-minute interval starting when the blood pressure measurement begins; A, 2-minute interval starting two minutes after the moment when the blood pressure measurement begins.
